# Application of artificial intelligence in nursing practice: a qualitative study of Jordanian nurses’ perspectives

**DOI:** 10.1186/s12912-024-02658-6

**Published:** 2025-01-25

**Authors:** Wesam Taher Almagharbeh, Hazem AbdulKareem Alfanash, Khaldoon Aied Alnawafleh, Amal Ali Alasmari, Faris Abdelkarim Alsaraireh, Mutaz M. Dreidi, Abdulqadir J. Nashwan

**Affiliations:** 1 Medical and Surgical Nursing Department, Faculty of Nursing, University of Tabuk, Tabuk, 7191 Saudi Arabia; 2 Department of Nursing Leadership and Education, Faculty of Nursing, University of Tabuk, Tabuk, 7191 Saudi Arabia; 3 Department of Community and Mental Health Nursing, Faculty of Nursing, Mutah University, Alkarak, Jordan; 4 Department of Nursing, Faculty of Pharmacy, Nursing and Health Professions, Birzeit University, Ramallah, Palestine; 5 Nursing Department, Hamad Medical Corporation, Doha, P.O. Box 3050, Qatar; 6 Department of Public Health, College of Health Sciences, QU Health, Qatar University, Doha, P.O. Box 3050, Qatar

**Keywords:** Artificial Intelligence, Nursing practices, Qualitative study, Thematic analysis, Jordan, Ethical concerns

## Abstract

**Background:**

Artificial Intelligence (AI) is increasingly applied in healthcare to boost productivity, reduce administrative workloads, and improve patient outcomes. In nursing, AI offers both opportunities and challenges. This study explores nurses’ perspectives on implementing AI in nursing practice within the context of Jordan, focusing on the perceived benefits and concerns related to its integration.

**Method:**

A qualitative research approach was employed, involving semi-structured interviews with 25 nurses and 3 focus group discussions, each consisting of 7–8 participants. The data collected was coded and analyzed using thematic analysis to identify recurring patterns and key themes in the nurses’ views on AI.

**Results:**

Three major themes emerged from the analysis: (1) AI as an efficiency tool – Nurses recognized AI’s ability to reduce administrative burdens and improve patient monitoring in real-time. (2) Ethical and practical concerns – Nurses raised issues regarding patient privacy, data security, and the fear that AI might replace human decision-making in care. (3) Lack of preparedness and training – There was a consensus on nurses’ inadequate training in AI tools, limiting their ability to integrate AI into their practice fully.

**Conclusion:**

While AI is seen as a valuable tool to enhance nursing productivity, several challenges still need to be addressed, particularly regarding ethical concerns and insufficient training. To ensure AI complements nursing without compromising the human element, healthcare institutions must address these issues by implementing comprehensive training programs and establishing clear ethical guidelines.

## Introduction

Artificial intelligence has been on the rise in the healthcare industry. It has been of great value in areas such as nursing, where it can enhance the efficiency of care provision and decrease the resistance quotient. In the broader health industry, the AI market has been projected to reach $45.2 billion by 2026, a year-on-year growth rate of 44.9% as it seeps into the clinical and operational spheres [1]. Now, AI is used in nursing to diagnose, monitor, and administer medications to patients. According to Sommer et al. [2], 25.2% of the nurses can be classified as AI expert nurses, while the results from Atalla et al. study [3] show that it is the nurses’ attitudes toward AI which significantly influence their innovative work behaviors, suggesting that favorable attitudes towards AI should help fostering integration of AI into nursing practice. It relieves the human nurses from handling everyday tasks that can be delegated to the AI so that the human nurses can concentrate on attending to the complicated patient needs [4].

However, incorporating AI in nursing has challenges. This paper sought to highlight, especially in poor-resourced settings such as Jordan. The healthcare system is burdened by a nurse-to-patient ratio far below the World Health Organization’s recommendation (1:14 vs. 1:6) [5]. In such contexts, nurses engage AI to reduce the burden on them; however, they raise issues of data protection, patient secrets, and automation of nursing jobs. More so, studies show that about 41% of the healthcare staff, including the nurses, needed more time to integrate artificial intelligence because of a lack of training and inadequate Artificial Intelligence machines [6, 7].

Artificial intelligence integration into nursing practice poses several challenges, including concerns about privacy of data because of requirements of health records, potential biases in the artificial intelligence algorithms that may lead to disparities in patient care, nurses need to get trained in a comprehensive way to serve new skills, and these new skill sets will require nurses to be trained intensively in order to develop them, and finally incorporation of artificial intelligence may demand heavy investment in technology and human resources [8]. Such challenges in part can be addressed by establishment or a re-instatement of robust data governance policies, development of ethical AI framework(s), targeted education and training of nursing professionals for implementing implementation of ethical AI framework(s) and moreover should adhere to clear guidelines of AI accountability and integration in health care system [9, 10].

The constraints due to ethical issues are further discussed as a major barrier to adopting AI in nursing, there is a considerable emphasis on algorithmic decision-making, increasing concerns about the role of analytics and human intelligence in highly charged care settings. For example, AI systems do not have human empathy as is the core of the nursing profession, thus, patient care is put at risk [11, 12]. Consequently, although the implementation of AI might bring benefits, it can only be introduced to the nursing work environment by handling the identified ethical, training, and cultural issues [13].

Building on these ethical and cultural challenges, understanding how nurses perceive and comprehend AI in their daily practice is critical for its successful integration. AI has the potential to support a workforce increasingly strained by demographic shifts and shortages of skilled professionals. However, as highlighted by a recent cross-sectional survey conducted among 114 nurses in Bavaria, Germany (67.5% female, 32.5% male), there is a significant knowledge gap. The survey revealed that only 25.2% of participants identified as AI experts, with many perceiving AI as merely computers (30%), programming-based software (25%), database tools (20%), learning systems (15%), or decision-making aids (10%). Despite 66.7% viewing AI as an opportunity, concerns about its uncontrollability and potential risks underscore the need for comprehensive training [14]. Addressing these knowledge gaps and apprehensions through targeted education is essential to empower nurses and foster confidence in leveraging AI responsibly within the ethical framework of patient-centered care.

For this research, Jordanian nurses’ attitudes towards AI are explored, focusing on the perceived advantages and disadvantages of the application in their workplace. The study encompassed a diverse cohort of nursing professionals, including registered nurses (RNs), licensed practical nurses (LPNs), certified nursing assistants (CNAs), critical care nurses, emergency room nurses, and nurse practitioners (NPs). This comprehensive inclusion aimed to capture a wide range of perspectives on artificial intelligence integration across various nursing roles.

The primary objectives of this study are as follows:

1. To assess Jordanian nurses’ attitudes toward AI integration in nursing.
2. To explore how AI can enhance nursing effectiveness by reducing administrative tasks and improving patient monitoring while addressing ethical concerns like patient privacy and job automation.
3. To evaluate nurses’ current knowledge of AI technologies and identify gaps in their training.
4. To propose practical strategies for healthcare organizations to implement AI as a supportive tool, maintaining the human element in nursing care.

## Literature review

### AI’s role in nursing care: opportunities and benefits

Some previous research evidences the implementation of AI in nursing care primarily as having a possibility of enhancing the efficiency of business processes and optimizing the outcomes of interventions with patients. According to Karim et al. [15], AI applications enable a decrease in administration workload to provide more time for patients. Their research focused on the fact that AI can reduce clerical tasks like patient administration and record keeping and, therefore, has the potential to improve patients’ outcomes.

According to Markus et al. [10], AI deals with time-consuming tasks like data analysis to ensure that nurses spend ample time with patients. Their work outlined the major opportunities for AI to contribute to the field of surgery and identified how AI might improve surgical hand-offs and promote the accuracy of the information exchanged with healthcare teams. This application ensures that it minimizes errors within compelling environments where accurate and real-time data is vital.

Martinez-Ortigosa et al. [16] also noted that AI consists of such functions, but nursing shall always remain relational, and AI should be incorporated in such a manner. In their systematic review of AI applications in nursing, they argued against complete automation of nursing tasks without reference to feeling and interpersonal relations inherent in nursing practice.

### Ethical and professional concerns

If AI is to be implemented in healthcare, cultural and ethical analysis will a center stage. Jordan et al. [17] observed that the management of culture has been identified as a common determinant of the success or failure of AI. The qualitative research on the use of AI in triage systems showed that practitioners of different cultural backgrounds experience varying degrees of trust in AI based on cultural values and encounter experiences. This study suggests it is high time to develop AI systems that respond to patients’ and clinicians’ cultural needs and expectations.

Regarding the nurses’ attitude towards AI, Alruwaili et al. [5] pointed out the positive attitude towards AI but concern about trust in such systems. Lichtenthal et al. also pointed out that confidence in AI decision-making among nurses was strongly tied to how the algorithm was explained and the level of explanation given to the AI. This transparency is necessary for the complete adoption of AI technologies.

Similarly, Castagno and Khalifa [13] noted that trust is the main component of the AI model. When asking healthcare staff, the survey’s authors showed that clinicians are ready to work with systems that reflect their values and provide reasons for operations. Therefore, the study established that for organizations to successfully adopt AI, issues related to reliability and trust must be addressed.

### The importance of training and preparedness for AI adoption

A key challenge of AI in nursing remains the inadequate training for AI tools. Kiger and Varpio [18] noted that while nursing personnel, for example, show interest in using AI, most need more technical capacity to realize this interest. Their guide to thematic analysis suggested that education, training, and other professional practice enhancements should be ongoing and specific to AI as this population requires basic training and booster courses to use AI interventions confidently.

Similarly, Martinez-Ortigosa et al. [16] also stressed the importance of education in incorporating AI. Their systematic review concluded that inadequate training limits nurses’ ability to utilize AI-enhanced technologies in care delivery; their results highlighted the need for initial and ongoing training to ensure effective AI integration within the nursing profession.

Sangers et al. [19] reflected these findings, and Seibert et al. [20] claimed that many nurses need to practice and train in applying AI in clinical areas. In their own mixed-methods study, they pointed out that the constituent elements of artificial intelligence should be incorporated into structured educational programs to explain its technical and ethical application for nurses.

Adding to this discourse, a meta-analysis by Amiri et al. [21] investigated the attitudes and knowledge of medical, dental, and nursing students toward artificial intelligence in healthcare. While their findings revealed that only 44% of the students possessed knowledge about AI, a significant 65% demonstrated a positive attitude toward its integration into healthcare practices. This underscores the urgent need to enhance education and training in AI not only for practicing nurses but also for students preparing to enter the field.

### Addressing AI’s limitations in healthcare settings

Nevertheless, like almost every concept, its drawbacks must be noticed, especially in intensive care. Sangers et al. [19] have pointed out that the diagnostic potential of AI can be contaminated by overreliance, particularly in such antiseptic specialties as skin cancer, where clinical decision-making skills are more sophisticated. AI was useful in diagnostic processes, according to their study, but the process requires human intervention to avoid chances of going wrong.

According to King et al. [9], AI has been discussed to increase the accuracy of perioperative hand-offs. However, they pointed out that AI’s performance differs in stressful and packed environments. The study mentioned that the development of AI tools should be subjected to rigorous testing when used in such environments because any error can compromise patient safety.

### Future directions and research gaps

The current literature proposes several themes to call for further research to enhance AI in nursing. Bohr and Memarzadeh [12] noted that despite notable interest in the short-term application of AI in diagnostics and office work, more needs to be understood about its long-term stability. From their review of papers on AI applications in healthcare, such as MYC, the authors opined that future research should determine the tools’ sustainability and ability to meet future healthcare needs, as shown in Table 1.

In the framework proposed by Adus et al. [1], patients and nurses were identified as important stakeholders in developing artificial intelligence. In their paper about patient engagement in AI development, they established that involving the users in the decision-making process at an early stage helps align the technology with ethical principles and fosters trust in the existing system among consumers.

Lastly, Bays et al. [11] highlighted the caution that the proposed future studies should focus on how AI could support human nursing care instead of taking over it. As shown in their clinical practice statement on AI in obesity management, although AI improves some parts of nursing, being human and personal with patients remains critical.

Kiger and Varpio [18] also noted a concern we share: the absence of follow-up research about AI’s effects. Their guide also suggested that much of the current work stays at the level of the shorter term, and much less is said about the long-term impact of AI on the roles or work of nurses themselves and the patients they serve. The present study addresses this gap in the knowledge of AI in healthcare by identifying important research questions.

**Table 1:** Comparative analysis of AI impact in nursing

Reference	Technique	Results	Limitations	Findings
Adus et al. [1]	Focus group discussions with patients	Patients supported AI but emphasized the need for transparency and trust	Focused solely on patients’ perspectives, neglecting providers’ views	Patients want early involvement in AI development to ensure ethical considerations are addressed
Alruwaili et al. [5]	Qualitative study (Interviews with nurses)	Nurses were aware of AI’s potential but expressed concerns about preparedness.	Did not explore specific AI applications relevant to nursing roles	Nurses require targeted education and trust-building strategies to adopt AI effectively
Fazakarley et al. [22]	Qualitative synthesis (Thematic analysis)	Identified recurring concerns about trust, transparency, and ethics	Limited primary insights due to reliance on secondary data	Trust in AI is critical for successful adoption in healthcare
Karim et al. [15]	Descriptive qualitative study (In-depth Interviews)	AI can alleviate administrative tasks, but concerns about job displacement	A small sample size may limit generalizability	AI should support nurses by reducing administrative burdens without replacing human care.
King et al. [9]	Qualitative study (Interviews with perioperative nurses)	AI improved perioperative hand-offs, enhancing accuracy	Focused only on perioperative care, missing other nursing contexts	AI can enhance specific workflows like perioperative hand-offs but requires further testing
Sangers et al. [19]	Qualitative study (Semi-structured interviews with dermatologists and GPs)	AI was useful for diagnostics, but over-reliance was a concern	Excluded nurses’ perspectives, limiting the comprehensiveness	Collaboration between AI developers and healthcare professionals is necessary for success
Seibert et al. [20]	Explorative sequential mixed-methods (Interviews, Surveys)	Ethical concerns need for training, and AI’s potential to improve care	Did not address AI’s long-term impact on nursing roles	Both technical skills and emotional readiness are crucial for AI adoption in nursing

## Methodology

### Research approach

This research used an exploratory, descriptive, and qualitative method, with semi-structured interviews and focus group discussions, to capture the nurses’ views on using AI in nursing practices. This approach was adopted because it enables the participants to give their opinions freely, and at the same time, the study was guided by the set research questions. The arrangement made it easy to maintain order in data collection while at the same time guaranteeing the discovery of similar themes among the participants. The focus groups allowed the sample to share their opinions within a group and for group processes to be observed, which may not be possible in individual interviews.

### Theoretical framework

The theoretical foundation for this research was anchored on the Technology Acceptance Model (TAM), which looks at how users adopt technology [23]. In the case of AI in nursing, the TAM was used to measure the perceived usefulness and ease of use of AI technologies from the nursing students’ point of view [24]. Moreover, critical views on ethical considerations concerning healthcare were explored regarding the bioethical principles of autonomy, beneficence, and non-maleficence [25].

The original Technology Acceptance Model (TAM) from Davis (1989) was used in the study. This model assesses acceptance of technology based on two primary factors: perceived ease of use and perceived usefulness. This foundational framework is then used effectively to evaluate nurses’ attitude towards artificial intelligence integration in healthcare settings [23].

### Data collection

Data were collected from September 10, 2023 to May 24, 2024 through semi structured interviews and focus group discussions with nurses in Jordan’s public and private healthcare facilities. Each semi structured interview lasted 50–60 min and the participant could answer freely while ensuring consistent topic coverage. The interviews were conducted in person or via video conference, as preferred by the participants, and were led with questions derived from a literature review; the guide was refined after five pilot interviews. As shown in Table 2, the interviews were complimented with focus group discussions which allowed the interactive dialogues among participants to disclose additional insights to collective perspectives and experiences.

**Table 2:** Summary of semi-structured interviews

Aspect	Description	Duration	Mode of Conduct
Interview Guide Development	Based on a comprehensive literature review piloted with 5 participants	N/A	Face-to-face and video conferencing
Interview Questions	Focused on understanding AI, experiences with AI, benefits, challenges, and recommendations for AI integration in nursing	50–60 min per session	Open-ended, semi-structured format
Pilot Test	Revisions made to the interview guide based on participant feedback	N/A	Conducted with 5 participants before the main study
Recording Method	Audio-recorded with participant consent, transcribed verbatim for analysis	N/A	Face-to-face or video conferencing, depending on preference

The interview questions included whether the nurses know about Artificial Intelligence, whether they used AI or came across it, the perceived benefits and drawbacks of case utilizing AI in nursing, and the potential AI application in the future nursing field. All interviews were conducted with the participant’s permission and recorded on audio for later transcription and analysis. Audio recording served the purpose of having a word-to-word account of the discussions and, in addition, verbatim transcription offered a textual account of the talk for the analysis.

### Sampling technique and sample criteria

A purposeful sampling technique was used to recruit registered nurses from different work areas, such as the intensive care units, outpatient departments, and general wards. The inclusion criteria were defined as participants’ employment in healthcare organizations that implemented or discussed AI technologies, ensuring their opinions aligned with the study goals. As shown in Table 3, the participants’ demographic and professional characteristics, such as gender, age, marital status, educational background, work experience, and employment type, were documented to provide a comprehensive profile.

**Table 3:** Characteristics of participants

Code	Gender	Age	Marital Status	Highest Degree	Working Experience (years)	Employee Type
NP1	Female	31	Married	BSN	8	Permanent
NP2	Female	33	Married	BSN,	7	Contractual
NP3	Male	38	Married	PhD	12	Permanent
NP4	Female	32	Married	MSN	6	Permanent
NP5	Female	30	Married	MSN	4	Permanent
NP6	Female	37	Married	MSN,	11	Contractual
NP7	Female	36	Unmarried	BSN	10	Permanent
NP8	Male	33.5	Married	BSN	7.5	Contractual
NP9	Female	29	Unmarried	MPH	3	Permanent
NP10	Female	28	Unmarried	MSN	5	Permanent
NP11	Male	41	Married	PhD	18	Permanent

BSN: Bachelor of Science in Nursing, MSN: Master of Science in Nursing, MPH: Master of Public Health,, PhD: Doctor of Philosophy

### Trustworthiness

Several measures were employed to enhance this qualitative study’s credibility, dependability, transferability, and conformability. First, member checking was used, whereby participants were given the transcripts of their interviews and a summary of the themes developed from the interviews to validate the data. Second, triangulation was employed by data source, using both semi-structured interviews and focus groups, and across various healthcare settings. This approach made the results more credible and consistent. Furthermore, the decisions made during data collection and analysis were recorded in an audit trail to increase the study’s credibility.

### Thematic analysis

The data was analyzed using thematic analysis, as [4] outlined. The analysis involved three main steps: Open, axial, and selective coding. The open coding included reading multiple transcripts to find significant statements and codes. In the next step, axial coding was done to integrate similar codes into categories, and, in the last step, selective coding was done to come up with themes that best captured the patterns in the data. This research employed NVivo 14 software in data management and coding which are tools that help in the management of large volumes of qualitative data systematically.

Two researchers also coded the transcripts for inter-observer reliability, discussing differences until a consensus was reached. The final themes were rechecked to capture the participants’ views appropriately.

### Ethical considerations

The Institutional Review Board (IRB) of participating healthcare institutions in Jordan approved ethical approval for the study. The study’s objectives, participants’ rights, and data confidentiality were explained to all participants. Interviews began with informed consent, and the participants were told they were free to withdraw from the study without penalty. The audio recordings and transcriptions were secure, and the names of the participants had been pseudonymized.

The rough methodology outlined above generates a robust and systematic way of thinking about how nurses view AI Integration in healthcare. This study employs a combination of semi-structured interviews, focus groups, and thematic analysis to provide a full understanding of the complex dynamics of AI adoption in nursing practice.

## Results

The results of this study are presented in three sections, corresponding to the techniques outlined in the methodology: They rely on semi-structured interviews, focus groups, and thematic analysis. This section presents findings of nurses’ perceptions of Artificial Intelligence (AI) integration in nursing practice in both public and private healthcare institutions in Jordan.

### Semi-structured interviews

Twenty-five nurses were interviewed semi-structured between September 2023 and May 2024. The interviews took about 50 to 60 min each, giving a total picture of what the nurses experienced, worried about, and recommended for AI in nursing. The results showed a mixture of optimism and concern about AI integration.

### Understanding of AI

Almost all participants were familiar with AI, describing it as a technology for administrative tasks, data analysis, and patient monitoring. However, nurses in private institutions demonstrated greater awareness of AI applications compared to those in public healthcare settings. Among the 25 participants, 18 had direct experience with AI systems in their workplace. Table 4 illustrates the participants’ understanding provided varied descriptions of AI, reflecting a range of familiarity and usage in their roles.

**Table 4:** Participants’ understanding of AI

Code	Understanding of AI
NP1	“AI is mainly for automating tasks like patient data entry.”
NP2	“AI helps with diagnosis, but I am unfamiliar with its use in nursing.”
NP7	“We use AI to monitor ICU patients, but I am still learning how it works.”
NP10	“AI is a tool that helps reduce our workload, especially with data handling.”

Participants from private institutions, such as NP7, were more familiar with AI in critical care environments. In contrast, participants from public institutions, such as NP1, were more familiar with AI as an administrative tool. This difference marks the variation in exposure to AI technologies, which reflects institutional resource and technology adoption.

### Perceived benefits of AI

As shown in Table 5, Participants widely recognized that AI would reduce nurses’ workloads, particularly in administrative and repetitive tasks. Twenty-one 25 participants applauded that AI freed them up to spend more time on direct patient care.

For example, NP5 stated:

“AI takes over the tedious data entry tasks so that we can focus on the patients’ needs.” — NP5.

Nurses said using AI-assisted monitoring systems, especially in critical care, yielded positive patient outcomes because real-time data analytics were provided. AI tools also helped many intensive care unit participants track patient data and glean insights, enabling more rapid, better-informed emergency decisions.

“AI helps us track patient data in real-time, and we can make decisions faster, especially in emergencies.” — NP9.

Although these benefits were well known, several participants pointed out that AI’s effectiveness hinges on how well it fits into their workflows. It was often mentioned that there was potential to reduce administrative burden and improve patient care quality.

To what extent should challenges be recognized? And confront the difficult ethical questions that emerge from it.

The semi-structured interviews also revealed great concerns about AI, especially ethical aspects. For example, 15 participants were concerned about patient privacy and data security, and 12 were worried that AI could cause job displacement.

**Table 5:** Participants’ challenges and ethical concerns

Code	Challenges and Ethical Concerns
NP3	“AI might affect patient privacy, especially with sensitive data.”
NP6	“We need more transparency in how AI systems make decisions.”
NP9	“AI could take over parts of our job, leading to fewer nursing positions.”
NP12	“AI may improve efficiency but cannot replace human empathy.”

Many participants also raised AI algorithm transparency. For instance, NP6 noted that AI systems need more clarity in making decisions, a common concern that AI may sidestep human judgment in critical care.

Participants also expressed concern about job displacement. NP9 commented that more tasks could be automated, and the fear is that AI could decrease the number of nursing positions. Participants spoke of AI’s potential but also pointed out the inability of AI to replace human empathy and emotional intelligence during patient care.

“AI may improve efficiency, but it cannot replace human empathy.” — NP12.

The semi-structured interviews showed that nurses in Jordan generally believed that AI could improve the efficiency of nursing tasks through automation and real-time data analysis. But the findings also raised serious questions about patient privacy, job displacement, and training and transparency of AI systems. Participants recognized that AI was not so much an enabler of more efficient workflows as it was an aid to getting things done (and perhaps less human things). However, it also confirmed that the human element of patient care cannot be eliminated and that AI technologies must be used ethically.

### Focus groups

Further, three focus groups of 7–8 nurses were conducted to broaden insights regarding nurses’ collective perspectives on integrating AI into nursing practices. The group dynamics in these sessions provided a richer discussion of shared concerns, insights, and experiences that might not have come out in individual interviews. The group discussions identified consensus on the potential benefits of AI and a great deal of concern over training, implementation, and ethical implications.

### Consensus on AI benefits

Individual interviews and focus group discussions were consistent with each other in that they recognized the possibility of using AI to improve efficiency in nursing. Repetitive tasks such as patient monitoring or data entry became one of the most frequently mentioned advantages. The participants agreed that AI could free up valuable time to allow nurses to spend more time on direct patient care, which they considered their first responsibility.

In all three focus groups, participants said that AI systems, particularly those used in ICUs and general wards, dramatically reduced the time spent on documentation. In describing how AI-assisted monitoring systems could alert nurses to critical patient changes and enable faster interventions, nurses described.

One participant from Focus Group 1 said:

“AI helps us focus more on patient care, our primary role. Instead of filling out forms, we get alerts when something critical happens with a patient, which helps us respond faster.”

Another participant from Focus Group 2 elaborated on the real-time advantages AI provides in emergency care settings:

“We use AI in our emergency department to track vitals and notify us of a significant change. It reduces human error and lets us act quickly when time is of the essence.”

Focus groups agreed that AI systems were practical, especially for reducing manual and time-consuming tasks. However, the discussions also made clear that the way AI is being used today is very much supplementary to human judgment, not replacing it. Nurses were generally satisfied with AI’s role in accelerating their efficiency in critical care and data management tasks.

### Concerns about training and implementation

While focus groups recognized AI’s potential benefits, a common theme remained in all focus groups: there needs to be more training and preparation for using AI technologies. However, participants thought AI could enhance healthcare practices with adequate education and hands-on experience. Many participants stated they needed to be adequately trained to use AI systems and were frequently unsure about what they could and could not do with these technologies.

Of the 25 nurses who participated in the focus groups, 19 mentioned insufficient training they received in their institutions. They called for more structured workshops and practical training sessions to help these nurses grasp what AI meant in their everyday work. Training programs, they said, should extend beyond theoretical knowledge to the practical use of AI in real-time clinical settings.

For example, one participant from Focus Group 3 stated:

“We need hands-on training to understand how AI fits into our daily work. Right now, it feels like an abstract concept. We’re using it without fully knowing how it works or how it can help us.”

Another participant from Focus Group 2 echoed similar concerns:

“AI systems are great, but I’ve never had a formal training session. I feel like I’m just figuring it out on the job, which isn’t ideal when dealing with patients’ lives.”

Several participants mentioned that AI technologies are growing rapidly, and we need to keep learning and taking refresher courses. They were concerned that their institutions’ training programs were not keeping up with technological advancement. They said ongoing professional development would be needed to keep up with AI’s role in healthcare on an ever-evolving basis.

### Implementation barriers and ethical concerns

A second key discussion area across the focus groups was how to make AI systems work. Some AI tools described by nurses promised some good things but needed to be completely integrated into existing workflows, causing inefficiencies and frustration. They pointed to cases in which AI systems were used in concert with manual processes, sometimes adding to rather than decreasing the work.

Participants in Focus Group 1 provided an example of an AI-based patient monitoring system that generated alerts but required manual data entry for updates:

“We get alerts from the AI system, but we still have to enter the data manually into the hospital’s main system. It feels like we’re doing double the work.”

In addition, these discussions raised ethical concerns about AI, particularly patient privacy and the accuracy of AI-driven decisions. Many nurses suggested they were uncomfortable relying too much on AI and that they found it uncomfortable when the system’s decision-making processes weren’t fully transparent. Everyone worried that AI would make mistakes in diagnosing or monitoring patients, which would be very dangerous.

A participant from Focus Group 3 commented on this issue:

“AI can help us a lot, but we don’t always know how it’s making decisions. If it flags something as urgent and wrong, we could treat patients unnecessarily.”

The discussions also highlighted a significant gap in understanding how AI handles sensitive patient data. Nurses expressed concerns that AI systems might compromise patient confidentiality, particularly if the systems were connected to broader hospital networks or cloud-based platforms.

“We need more information on how patient data is stored and who can access it. With AI systems, there’s always a risk that sensitive information could be exposed.” — Participant from Focus Group 2.

### Summary of focus group

The focus groups provided a collective perspective on AI integration in nursing, reinforcing many themes from the semi-structured interviews. Participants across all groups acknowledged the potential benefits of AI in improving efficiency and reducing workload but emphasized the need for comprehensive training to maximize these benefits. Concerns about implementing AI technologies, particularly regarding workflow integration and ethical considerations, were also prevalent.

**Table 6:** Key insights from focus group discussions

Focus Group	Key Insights
Focus Group 1	AI improves efficiency but needs to be better integrated with manual systems to avoid doubling the workload.
Focus Group 2	Nurses require structured, hands-on training to fully understand AI’s potential and how it fits into their daily responsibilities.
Focus Group 3	There are significant concerns about the ethical implications of AI, particularly regarding patient privacy and decision-making transparency.

Table 6 summarizes these insights, highlighting the unique contributions of each focus group while reflecting shared concerns. For instance, Focus Group 1 emphasized the importance of seamlessly integrating AI with manual systems to avoid doubling the workload. Focus Group 2 stressed the need for structured, hands-on training to help nurses fully understand AI’s potential and its fit into daily responsibilities. Meanwhile, Focus Group 3 raised significant concerns about ethical implications, particularly related to patient privacy and decision-making transparency. Overall, the focus groups agreed that AI needed proper education and implementation strategies to truly live up to everyone’s hopes for it by being used by nursing. The nurses expressed optimism and caution and spoke of AI opportunities, but all said AI needed to be embedded in a way that enhances their work while ensuring the safety of their patients.

### Interviews and focus group thematic analysis

Through thematic analysis, recurring patterns across semi-structured interviews and focus groups were identified. This process revealed three prominent themes: Efficiency, Ethical and Practical Challenges, and the Need for Training and Education—AI as an Enabler. These themes capture the ambiguities of nurses’ attitudes and encounters with Artificial Intelligence (AI) as it integrates into their daily work. Table 7 presents these main themes, sub-themes, participants’ quotes, and codes, which highlight both the benefits and concerns related to AI integration in nursing practice.

**Table 7:** Main themes, sub-themes, participants’ quotes, and codes

Main Theme	Sub-themes	Participants’ Quotes	Codes
AI as an Enabler of Efficiency	Administrative task reduction	“AI helps reduce the time spent on paperwork so that we can focus on patient care.” (NP5)	Efficiency
Ethical and Practical Challenges	Privacy and job displacement	“I worry about AI taking over some of our roles, and also about how patient data is handled.” (NP9)	Ethical concerns
Need for Training and Education	Lack of AI training	“We need structured training to understand how to use AI in nursing.” (Focus Group 2)	Training gaps

### Theme 1: AI as an enabler of efficiency

The analysis revealed that overall, nurses considered AI a major tool to boost the efficiency of nursing tasks. As a theme, it was found in both interviews and focus groups. People also pointed out that AI has cut down the time required for doing mundane robotic administrative work like entering patient data, creating reports, and how to track a patient’s progress. In turn, it has enabled nurses to spend more time focusing on direct patient care, which is the essence of their job. In critical care units where real-time monitoring is necessary, AI helps many nurses streamline their workflow.

AI-enabled manual data handling errors sped up tasks such as processing patient vitals and staff alerting to critical changes and reduced manual data handling errors. However, the literature lacks insights from intensive care unit (ICU) nurses on how AI use in patient monitoring systems improved the time to detect abnormal trends in vital signs, which may avert critical incidents. With AI implemented consistently in routine monitoring, there was a smoother workflow and faster response time.

However, there was a feeling that AI systems needed to be fully leveraged throughout all departments. Public institution nurses said AI had a lot of potential, but the systems needed to be developed or integrated better to take advantage of it. Many participants said that if AI were more widely and cohesively adopted, it would enable them to manage time and resources better and, in turn, improve patient outcomes.

### Theme 2: ethical and practical challenges

Participants acknowledged AI’s role in improving efficiency but repeatedly raised ethical and practical challenges. Among the greatest concerns raised were the privacy of the patient and how sensitive AI systems handle data. Participants were worried about the security protocols for AI systems, worrying that patient data could be breached. Nurses were worried about how data is stored and accessed, and they all wanted to know more about how algorithms and processes work behind AI’s decision-making.

They worried that nurses working in settings where AI was more commonplace could be displaced. They were aware that AI could help with many of their jobs, but they were also very worried that as AI gets smarter, it will replace humans in making decisions. Participants insisted that AI should supplement, but not substitute, human judgment and care. Across different settings, there was the fear that AI would take over the personal, empathetic nature of nursing.

But many nurses also said that despite its support for diagnostic processes and patient monitoring, AI simply didn’t have the emotional intelligence or human empathy needed for patient care. It was found that for nursing to be much more than clinical care, the work includes the emotional needs of the patients. Everyone agreed that AI could not replace this most basic aspect of nursing and should not be programmed to do so.

### Theme 3: need for training and education

Another recurring theme in the interviews and focus groups was the need for structured, comprehensive training. Nurses said they needed to prepare to use AI effectively, lacking formal training and educational resources. While AI was being used in their institutions, most participants believed they needed to be sufficiently guided on using these tools daily. Nurses were given limited training and often had to learn how to use AI applications through trial and error.

Participants wanted hands-on training sessions where they could learn more about AI systems. They said that understanding their capabilities, limits, and inner workings would make them more efficient and safer for patients. A popular and clear need was for ongoing professional development programs meant to update nurses on the latest advances in AI and ensure their confidence in using these systems.

Several also pointed out that AI technology is rapidly evolving, and training is ongoing rather than a one-time event. Nurses hoped that with regular refresher courses, they might fall asleep and be behind AI systems as they continue to develop, compromising the quality of the care nurses provide.

### Summary of thematic analysis

The thematic analysis results suggested the nature of this complex relationship between nurses and AI in clinical practice. However, AI was well recognized as a means to make nurses more efficient by automating their administrative tasks and boosting data management accuracy. Yet it’s far from settled, regarding data security and ethics of AI, and the fear that AI will replace nurses in roles. The urgent need to train more comprehensively and continuously became a key factor in successfully integrating AI into nursing practice. Thematic analysis of nurses’ relationships with artificial intelligence (AI) in clinical settings indicates a complex relationship. AI is generally acknowledged to be something that will help make nurses more efficient by automating administrative tasks and improving data management accuracy. But there are fears that there isn’t enough thought being given to the security of the data, ethical implications and whether or not AI will usurp nursing roles. Because of these issues, successful incorporation of AI into nursing care requires comprehensive and continuous ongoing training. In this study, we underscore that ethical standards in nursing need to be upheld while technological advances that enhance nurse’s capabilities, rather than stand in its place.

### Research techniques comparative analysis

This study employed three qualitative research techniques: focus groups, semi-structured interviews, and thematic analysis. Each method was unique in its contribution to understanding nurses’ perspectives on AI integration in nursing practice. The following sections compare these techniques in detail and highlight their strengths, limitations, and contributions to the research. Table 8 presents a comparative analysis of the research techniques, emphasizing their respective strengths, limitations, and key contributions to the study.

**Table 8:** Comparative Analysis of Research Techniques

Technique	Strengths	Limitations	Key Contributions
**Semi-Structured Interviews**	- Captured rich, in-depth, and personalized responses. - Allowed follow-up questions to delve deeper into specific areas. - Facilitated detailed exploration of individual experiences, both positive and negative.	- Time-intensive, requiring significant effort for conducting interviews and transcribing responses. - Limited to individualized perspectives, lacking the broader group dynamics that provide a collective understanding.	Provided detailed insights into personal experiences and individualized concerns, including nuanced perspectives on AI’s impact in nursing practice.
**Focus Groups**	- Fostered interactive discussions and group dynamics, enabling participants to build on each other’s responses. - Facilitated the identification of collective concerns, such as training gaps and job security. - Highlighted consensus on AI’s perceived benefits.	- Group settings may inhibit some participants from fully expressing their views, particularly if they feel overshadowed by dominant voices. - Risk of groupthink or bias influenced by more vocal participants.	Offered insights into shared challenges and collective attitudes toward AI, highlighting the importance of consensus-building and group-oriented strategies.
**Thematic Analysis**	- Synthesized data from both interviews and focus groups, ensuring a holistic view. - Provided a systematic approach to identifying recurring patterns and generating comprehensive themes.	- Dependent on the richness and quality of collected data. - Coding and categorization processes were time-intensive and required meticulous attention to detail.	Enabled the creation of overarching themes like efficiency, ethical concerns, and training needs, contributing to a structured understanding of nurses’ perspectives on AI.

Combining semi structured interviews and focus groups enabled a thorough exploration of nurses’ attitudes to AI. Interviews gave us deep individual insights, focus groups made us share collective sentiment and bring us diverse perspectives. These qualitative data were effectively synthesized by thematic analysis with themes circling around efficiency improvements, ethical considerations, and were required to have comprehensive training. While the methods are time intensive and require participant openness, careful planning and skilled moderation are required to limit the potential biases and to obtain rich, reliable data collection.

The integration of AI in nursing practice from the nurses’ point of view was explored using a combination of semi-structured interviews, focus groups, and thematic analysis. Semi-structured interviews provided detailed personal insights about individual experiences, while the focus groups helped bring out the group dynamics and shared challenges and perspectives. The thematic analysis synthesized findings from both techniques to elicit key themes that included the role of AI in increasing efficiency, ethical and practical issues, and the overall need for comprehensive training.

While each method had strengths and weaknesses, they offered a multi-faceted approach. A deeper exploration of personal views was possible through semi-structured interviews, group consensus, and shared challenges were emphasized through focus groups, and thematic analysis provided a structured framework to understand overarching patterns across all data sources. Combining this approach allowed the study to reflect on individual and collective insights. It helped deliver a balanced and all-inclusive picture of how AI is being integrated into nursing practice.

## Discussions

This study finds that Jordanian nurses have a complex but generally optimistic view of integrating Artificial Intelligence (AI) in nursing practice. However, this optimism is tempered by serious issues of ethics, job displacement, training deficits, and practical implementation problems. Results echo previous studies while offering new insights into the context-specific challenges of AI adoption in Jordanian healthcare, especially for nursing.

### AI as a tool for efficiency

In the present research, participants attributed many benefits of AI to increasing the efficiency of the nurses through decreasing form fillings and providing more effective, real-time patient surveillance. This is consistent with findings indicated by Karim et al. [15], where authors noted that AI applications reduce clerical burdens to enable nurses to attend to more patients. Al-Sabawy [4] also concluded that AI automation saves time and downstream effort enabling more added-value care, according to the studied participants.

Fazakarley et al. [22] also highlighted the effects of infusing Artificial Intelligence on enhancing the hand-off procedures within the operative setting more accurately and with a shorter time for decisions in the ICU. However, in the present study, several Jordanian nurses pointed out that AI systems must be more advanced and integrated into their practice environments, leading to defects rather than effectiveness. This agrees with Castagno and Khalifa [13], where the authors pointed out that ineffective user interfaces and limited incorporation into the nursing processes result in additional work rather than making processes faster. For that reason, the acclaimed opportunities for amendments to the nursing practice concerning the application of AI have to be enhanced by enhancing the compatibility of the systems in question with the existing practices and introducing AI systems into the healthcare setting.

### Ethical and practical concerns

Concerns about ethical issues were identified as major themes in this study, including data privacy and security and the potential loss of jobs due to the implementation of AI. Similar feelings are described comprehensively in the literature. However, Nash et al. [26] pointed out that healthcare professionals in Ontario feared that AI decision-making was not transparent, leading to distrust. Similarly, in this study, the interviewed Jordanian nurses expressed concern regarding the AI’s black box nature; the nurses everywhere, including in the intensive care unit, feared that AI could make some lethal mistakes. This tallies with Sangers et al. [19], who pointed out that the participants’ concerns involved the risks associated with leaning on the algorithm too much when diagnosing patient conditions, as human supervision of the AI-built models is critical.

Also, there were quite high expectations of massive job losses due to the extension of automation through AI. Adus et al. [1] studied this question and found that healthcare workers were rigid about using artificial intelligence to alter human-centered decision-making in healthcare provision. This work affirms that Jordanian nurses entertain such fears, especially about AI’s ability to do away with aspects such as the human touch and emotions. Interestingly, even though AI may enhance some tedious and time-consuming nurses’ tasks, it has no human touch, relationship, or feelings related to patients’ needs that are crucial to the care processes, according to Seibert et al. [19]. Hence, all the appreciation for what AI can do for the nursing process should continue the need for the human touch in caregiving.

### Training and preparedness

One of the major implications of this research is that nurses need to be sufficiently trained to implement AI technologies, making them uncomfortable in their usage. This is in line with the study by Alruwaili et al. [5] that although the nurses know the benefits of using AI, they need more competence to incorporate it because of inadequate training. Kiger and Varpio [18] also found that most nurses need to be better equipped with knowledge and exposure on how to utilize AI systems safely and effectively to their optimum to enhance patient care.

Over and over, this study points to the lack of structured professional development programs to support teachers in this regard. Suppose these nurses fail to attend training sessions regularly. In that case, they may find themselves in a position where they cannot efficiently use AI in their practice, which would reduce the benefit that a particular implementation can bring in the event of an outbreak. According to Martinez-Ortigosa et al. [16], the need for related training and education is one of the biggest challenges when implementing AI. Improved continuing education efforts will help nurses be equipped to use AI better and decrease the stress related to its adoption.

### Cultural sensitivity and trust

In this study, cultural issues and trust were identified as very essential factors that would determine AI uptake. Nurses outlined the issues regarding the cultural relevance and trustworthiness of the systems, which correlates with Jordan et al. [17], who claimed that culturally integrated approaches can help gain trust in AI systems. Some of the participants in this study reported their reluctance to fully adopt AI based on the existing lack of trust in AI and its developers.

In addition, Bohr and Memarzadeh [12] highlighted the importance for the developers of AI and the healthcare providers to develop trust to implement AI. Participants’ views outlined that a partnership between AI developers and nurses should be fostered to allow the convergence of AI’s practical and ethical use in nursing. Such a discovery shows that the first-order involvement of nurses is a worthwhile necessity to enhance the process of AI system design and utilization in healthcare organizations.

### Constraints of geopolitical aspect

Artificial Intelligence (AI) and the integration of AI into the healthcare sector depends highly on its existing geopolitical context in Jordan. The regional instability and the attendant refugee influx has put stress on healthcare resources, taking funds away from technological advancements such as AI. Jordan has become the host of over 3 million migrants, about one third of the population, putting huge burden on the healthcare services by 2024. Constraints on investments in AI systems and training programs are further economic in nature and healthcare expenditure contributes 8.4% of the 2016 Gross Domestic Product (GDP) [16, 21].

Despite initiatives such as the Hakeem program to digital transform healthcare, political instability impedes long term consistent planning. Furthermore, nurse education and training disparities prevent many nurses from being adequately prepared to use and interpret AI effectively — 70.9 per cent of nurses had basic knowledge of AI, but further training of healthcare professionals is needed to improve AI literacy in healthcare [21]. These constraints are barriers to AI adoption and emphasize the necessity of targeted investments and capacity building to make AI’s potential in resource constrained settings as powerful as possible.

## Conclusion

This study explored the perspectives of nurses in Jordan regarding integrating Artificial Intelligence (AI) in nursing practices, using semi-structured interviews, focus groups, and thematic analysis. The findings revealed that AI is generally seen as an enabler of efficiency, particularly in reducing administrative tasks and improving real-time monitoring in critical care settings. However, the study also highlighted significant concerns, particularly related to ethical and practical challenges, such as patient privacy, data security, and the potential for AI to displace human roles. Moreover, the need for comprehensive training emerged as a recurring theme, as nurses felt they needed to prepare to leverage AI technologies fully. While AI holds promise for enhancing nursing workflows, its successful integration depends on addressing these concerns and ensuring that the human aspect of nursing care is preserved. Combining individual and collective perspectives, this study offers a holistic view of AI’s opportunities and challenges to nursing. This study also showed that Jordanian nurses generally regard the incorporation of Artificial Intelligence (AI) into nursing practice with cautious optimism.

### Implications of the study

The study has several important implications for healthcare institutions and nursing practices:

- **Enhanced Efficiency in Nursing Practices**: AI is promising a way to make administrative tasks easier so nurses can focus on patient care. This could achieve more efficient workflows and better patient outcomes in high-pressure environments like ICUs and the ED.
- **Ethical Considerations in AI Deployment**: There are some big ethical questions around the use of AI in healthcare institutions, as today’s patients must prioritize the ethical use of AI in their medical care: patient data privacy and data security. However, ethical guidelines should be set that clearly explain to nurses how and when certain decisions will be made; this fails to guarantee that the AI systems will be implemented without any background.
- **Investment in Training and Education**: It shows how crucial those programs are to building nurses’ skills and knowledge to use AI most effectively. Nurses must be trained to stay on par with technological developments, and institutions should offer professional development for nurses as added insurance.
- **AI as a Complement to Human Judgment**: In other words, AI should be thought of as a tool for enriching human judgment, not a tool of replacement. Healthcare providers say AI systems should be designed to allow nurses to deliver compassionate, patient-centered care while maintaining the essential human aspects of nursing, such as empathy and communication.
- **Collaboration between AI Developers and Healthcare Providers**: AI developers need to work closely with healthcare practitioners to develop AI systems that satisfy nurses’ practical needs and seamlessly integrate them into existing workflows. User-friendly interfaces and tools that improve nursing capabilities should be prioritized.

### Future work

Therefore, the continuous effects of the integration of AI into the nursing processes and the benefits on the patients’ care delivery should be examined in future research. This would be different for future research topics that examine how AI can contribute to decision-making in nursing, beyond mere administrative procedures into the possible potential of AI in nursing. Moreover, research involving multiple nations to look at the differences and comparisons between multiple healthcare systems that incorporated the use of AI can reveal details of the restrictive or enabling structural and cultural nature of the various institutions involved in the push, acceptance, and implementation of AI. Another promising avenue for future research would be looking not only into the efficiency of applying AI in communication with patients with cancer but also into such aspects as improving empathy.

### Recommendations

Based on the findings of this study, several recommendations can be made to improve the integration of AI in nursing practices:

- **Comprehensive Training Programs**: To promote safer environments for AI implementations in healthcare institutions, precise and practical training theories for nurses are recommended, with an emphasis placed not only on the practical aspect of using AI but also on the abilities of the nurses on such issues as limitations, possible ethical problems and opportunities of the application of AI. Professional training should be made to allow nurses to update themselves with current technological developments, such as AI.
- **Ethical Guidelines for AI Use**: AI development and applications should have rules that will prevent the misuse of patient data and lack of transparency in AI-driven decisions. For this reason, institutions that develop such guidelines should consult healthcare professionals primarily to identify issues of concern to nurses.
- **Collaboration between AI Developers and Healthcare Providers**: There should be a discussion between the technical creators of artificial intelligence and the healthcare organization to ensure that the AI systems are created to address the working world of nurses. Solutions should be developed where users are addressed through interfaces that do not interfere with nursing work and carry out data analysis in real-time and effective support tools.
- **Patient-Centered AI Development**: The application of AI systems proposed in designing care delivery should be more focused on enhancing the anthropomorphic characteristics of care, especially empathy and patient-nurse interaction. In this suffering, AI should help to make caring communication by nurses much more personalized and compassionate instead of emphasizing primarily productivity.

## Appendix

**Table:** Appendix: interview and focus group guide

Section	Semi-Structured Interview Guide	Focus Group Discussion Guide
Introduction	- Brief study introduction and purpose. - Explanation of confidentiality and voluntary participation. - Consent confirmation for recording.	- Welcome and overview of discussion topic. - Explanation of group dynamics, confidentiality, and voluntary participation. - Consent confirmation for recording.
General Questions	- Describe your role in nursing and your professional experience. - Have you encountered or used AI technologies in your workplace?	- What comes to mind when you think about AI in nursing?
Perceptions of AI	- How do you define AI in the context of nursing? - What are the potential benefits of AI in your field? - Do you see any risks or challenges?	- How do you perceive the role of AI in improving nursing care? - Are there areas where AI could have a significant impact?
AI and Work Efficiency	- How could AI help reduce your workload? - Examples of tasks AI could perform to assist you.	- How do you think AI can enhance nursing efficiency and productivity?
Ethical Concerns	- Are you concerned about patient privacy or data security when using AI? - Thoughts on AI replacing human roles.	- What ethical concerns do you associate with AI, such as decision-making and confidentiality?
Training and Preparedness	- Have you received any training related to AI technologies? - What resources would you need to feel more confident using AI?	- What kind of training do nurses need to use AI effectively?
Future Outlook	- What is your opinion on the future of AI in nursing? - Suggestions for preparing nurses for AI adoption.	- In one sentence, what would you recommend for integrating AI into nursing practice?

## Data Availability

The datasets are available from the corresponding author upon reasonable request.
